# A Risk-Stratification Machine Learning Framework for the Prediction of Coronary Artery Disease Severity: Insights From the GESS Trial

**DOI:** 10.3389/fcvm.2021.812182

**Published:** 2022-01-18

**Authors:** Nikolaos Mittas, Fani Chatzopoulou, Konstantinos A. Kyritsis, Christos I. Papagiannopoulos, Nikoleta F. Theodoroula, Andreas S. Papazoglou, Efstratios Karagiannidis, Georgios Sofidis, Dimitrios V. Moysidis, Nikolaos Stalikas, Anna Papa, Dimitrios Chatzidimitriou, Georgios Sianos, Lefteris Angelis, Ioannis S. Vizirianakis

**Affiliations:** ^1^Department of Chemistry, International Hellenic University, Kavala, Greece; ^2^Laboratory of Microbiology, School of Medicine, Aristotle University of Thessaloniki, Thessaloniki, Greece; ^3^Labnet Laboratories, Thessaloniki, Greece; ^4^Laboratory of Pharmacology, School of Pharmacy, Aristotle University of Thessaloniki, Thessaloniki, Greece; ^5^First Department of Cardiology, AHEPA University General Hospital of Thessaloniki, Thessaloniki, Greece; ^6^School of Informatics, Aristotle University of Thessaloniki, Thessaloniki, Greece; ^7^Department of Life and Health Sciences, University of Nicosia, Nicosia, Cyprus

**Keywords:** personalized (precision) medicine, coronary artery disease, machine learning, SYNTAX score, risk-stratification model

## Abstract

Our study aims to develop a data-driven framework utilizing heterogenous electronic medical and clinical records and advanced Machine Learning (ML) approaches for: (*i*) the identification of critical risk factors affecting the complexity of Coronary Artery Disease (CAD), as assessed via the SYNTAX score; and (*ii*) the development of ML prediction models for accurate estimation of the expected SYNTAX score. We propose a two-part modeling technique separating the process into two distinct phases: (a) a binary classification task for predicting, whether a patient is more likely to present with a non-zero SYNTAX score; and (b) a regression task to predict the expected SYNTAX score accountable to individual patients with a non-zero SYNTAX score. The framework is based on data collected from the GESS trial (NCT03150680) comprising electronic medical and clinical records for 303 adult patients with suspected CAD, having undergone invasive coronary angiography in AHEPA University Hospital of Thessaloniki, Greece. The deployment of the proposed approach demonstrated that atherogenic index of plasma levels, diabetes mellitus and hypertension can be considered as important risk factors for discriminating patients into zero- and non-zero SYNTAX score groups, whereas diastolic and systolic arterial blood pressure, peripheral vascular disease and body mass index can be considered as significant risk factors for providing an accurate estimation of the expected SYNTAX score, given that a patient belongs to the non-zero SYNTAX score group. The experimental findings utilizing the identified set of important risk factors indicate a sufficient prediction performance for the Support Vector Machine model (classification task) with an *F*-measure score of ~0.71 and the Support Vector Regression model (regression task) with a median absolute error value of ~6.5. The proposed data-driven framework described herein present evidence of the prediction capacity and the potential clinical usefulness of the developed risk-stratification models. However, further experimentation in a larger clinical setting is needed to ensure the practical utility of the presented models in a way to contribute to a more personalized management and counseling of CAD patients.

## Introduction

Nowadays, the advances in -omics disciplines and nanotechnology provide new clinical and therapeutic directions in the healthcare environment. The concept of personalized (precision) medicine is based on the exploitation of the molecular knowledge in the clinical practice so that the practitioners are able to evaluate more precisely prognosis and diagnosis of illnesses, as well as to deliver therapeutics. By working toward the establishment of precision medicine, however, scientists and clinicians face several challenges in healthcare. This difficulty deals with the attempts to achieve practical clinical utility by exploiting a diverse source of information, knowledge, and methodologies. Moreover, the added value and the implementation of the generated molecular knowledge into the clinical setting to guide personalized therapeutic decisions for patient populations, and/or individual patients is still challenging (1–4). The latter can be achieved through the introduction of *Machine Learning* (ML) prediction models that are adjustable and accountable the molecular heterogeneity of biological systems in real-time, present accuracy and reproducibility in the clinical setting, as well as aim to achieve broad clinical utility compliant to regulatory issues worldwide (5).

The evaluation of angina equivalents remains an intractable problem in symptomatic patients with suspected coronary artery disease (CAD). In clinical practice, CAD is routinely diagnosed via invasive angiography and non-invasive functional tests. Although these procedures decrease the misdiagnosis of stable CAD, they do not always seem to be necessary and can sometimes be considered as an excessive medical approach (6). Therefore, models and risk scores, which could accurately predict the pre-test probability (PTP) of obstructive and severe CAD in patients with suspected CAD, could facilitate the selection of patients who would benefit most from further diagnostic assessment or invasive treatment (7).

Several trials have been conducted to pool novel risk models for the effective risk-stratification of patients with suspected CAD. The model proposed by Diamond and Forrester (8) was the first and most widely used; yet it appeared to overestimate PTP for obstructive CAD and was therefore updated to also include age, sex, and symptoms as further predictive indicators (9). However, a “*battle of scores*” predicting PTP has followed over the last decade with many trial-based risk models emerging up-to-date (10–12), but their integration into clinical practice remains limited due to their inability to accurately predict the extent of CAD. Therefore, the fundamental question of whether the developed scores remain clinically useful or should be updated in contemporary populations remains unsolved.

Being motivating by the above considerations, the aim of the present study is to develop a data-driven framework based on ML approaches and the electronic clinical and medical health records collected from a retrospective cohort analysis of patients with suspected CAD undergoing coronary angiography. The purpose of the data-driven framework is twofold: (*i*) to identify a set of critical risk factors associated to the severity of CAD; and (*ii*) to build data-driven ML predictive models for the detection of symptomatic patients with severe CAD. To meet our objectives, we propose a two-stage modeling approach that combines both classification and regression ML techniques into a unified risk-score assessment process. In the first step, the focus is on distinguishing patients into two groups indicating patients presenting a zero- or non-zero SYNTAX score, whereas in the second step, the objective is to provide an estimate of the expected SYNTAX score for patients categorized into the non-zero SYNTAX score group. The proposed approach resulted in a two-part model that is able to adequately handle the excess of zeroes presented in the SYNTAX score distribution causing serious implications on the deployment of traditional statistical modeling techniques. Moreover, it is enhanced by a feature selection ML technique that provides a simple way to identify a set of potentially different important risk factors that affect the output of the two separated modeling processes. This capability leads to better understanding of the set of medical and clinical features that are related to: (*i*) the zero/non-zero SYNTAX score; and (*ii*) the strictly positive SYNTAX score distribution.

The proposed framework aims to provide a unified risk-score assessment able to identify low-risk patients in need of either anatomical or functional non-invasive testing to evaluate suspected CAD, and patients with complex CAD in need of urgent coronary revascularization procedure, thereby enabling a more personalized approach in the every-day clinical routine through the development of automated recommendation systems, based on data-driven perspective analytics algorithms.

## Methodology

In this section, we present, in detail, the methodology followed throughout the study in order to meet our objectives.

### Study Design

The current study is a *post-hoc* analysis of the GESS trial (ClinicalTrials.gov, Identifier: NCT03150680), the protocol of which has been already published elsewhere (13). GESS was a prospective, non-interventional cohort trial enrolling patients who underwent scheduled or emergency coronary angiography in the tertiary academic hospital of AHEPA, in Thessaloniki, Greece. In brief, this trial aimed to develop a novel risk prediction algorithm facilitating the prediction of the complexity of CAD, based on a large panel of genetic markers, combined with clinical and angiographic characteristics.

### Study Population

The population of this study consists of 303 adult patients with suspected stable angina enrolled in the GESS study. Patients were eligible for this analysis only if they presented with pain, suggestive of stable angina pectoris: typical, atypical, or non-specific chest pain. Patients were excluded if they met one of the following criteria: (*i*) known history of CAD; (*ii*) presenting with acute coronary syndrome (ACS); (*iii*) history of previous revascularization procedure (percutaneous coronary intervention (PCI), or coronary artery bypass graft surgery (CABG); and (*iv*) severe comorbidity with a life expectancy <1 year or cardiopulmonary arrest at presentation (see Supplementary Material).

### Ethics

This study was conducted in compliance with the declaration of Helsinki (14). The GESS trial has been approved by the Scientific Committee of AHEPA University Hospital (reference number 12/13-06-2019). Written informed consent has been provided by every participant of the study prior to their enrolment.

### Proposed Framework

**Figure 1:**
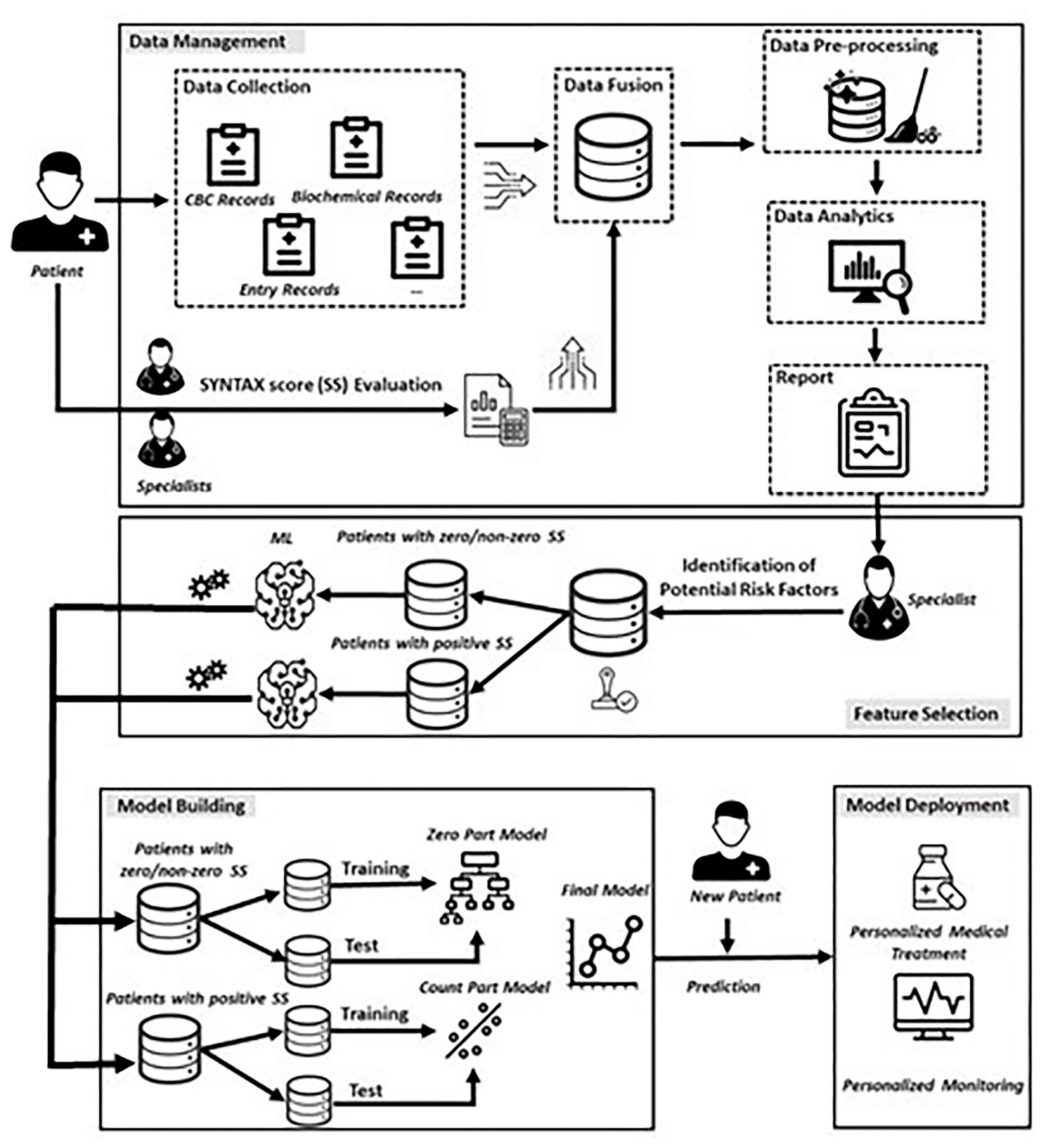
Proposed data-driven framework.

In this section, we present the proposed data-driven approach (Figure 1) consisting of four phases that are (*i*) *Data Management*, (*ii*) *Feature Selection*, (*iii*) *Model Building*, and (*iv*) *Model Deployment*.

#### Data Management

The first phase involves the collection of relevant data from the existed heterogenous sources related to the medical history and clinical examination of each patient, whereas two expert cardiologists evaluated independently the SYNTAX score of each patient enrolled in this study. Any possible conflict in the SYNTAX score assessment was resolved by a third expert. This step resulted to a unified database, in which all raw data were finally, aggregated and stored in appropriate data format.

The next step involves the careful investigation of raw data for deciding, whether they fulfil the quality criteria of the task being executed, since the extraction of meaningful insights depends heavily on the collected data. To this regard, a series of pre-processing techniques (*feature encoding, correction and removal of inconsistent values, missing value handling* etc.) were performed, resulting, in turn, into the final dataset.

After the preparation and finalization of the dataset, appropriate data analytics techniques were performed on the full set of the collected features. To this end, we made use of appropriate univariate descriptive statistics methods and visualization techniques for summarizing the characteristics of patients, whereas exploratory analysis was also applied for identifying potential effects of risk factors on the SYNTAX score distribution. The results of these two types of analysis were documented into a report for initiating a round of discussion with cardiology experts with the aim of identifying a preliminary set of candidate features that would be meaningful for participating into the building of the ML models.

#### Feature Selection

Although the utilization of the entire set of collected features could be a reasonable choice for building a ML model, this policy increases the dimensionality of the data with possibly irrelevant, noisy, or redundant features. Moreover, in medical applications, health-care experts are interested in understanding the mechanisms related to the variable of interest, rather than using a high-dimensional set of features resulted from a black-box process. Thus, the approved by the expert cardiologists subset of risk factors was the main input of a ML feature selection mechanism for deciding upon the final set of risk factors with a significant effect on SYNTAX score response. To this regard, we utilized the *Boruta* algorithm which is a wrapper method built around the random forest algorithm (15). The rationale behind this approach is the exclusion of irrelevant features that are proved to be less relevant to randomized versions of them. More specifically, the Boruta algorithm consists of the following seven steps (15), and was separately executed for two specific subsets of patients (more details are described into the following section):

1. Duplicate the initial dataset by creating a copy of each independent variable and add randomness to the new generated variables (called *shadow features*) by shuffling (permuting) their raw values.
2. Train a random forest model on the extended dataset and evaluate the importance of each feature (both the original and shadow features) using a pre-defined measure of importance (e.g., *Mean Decrease Accuracy*).
3. Evaluate the *z*-score of each original feature and compare it with a threshold defined as the *maximum* *z-score among shadow features* (MZSF).
4. For each feature presenting a *z*-score lower (or higher) than MZSF record this hit (0/1) in a vector.
5. Repeat the process for a predefined set of iterations.
6. Use the binomial distribution to assess whether each original feature is characterized as “*non-informative*,” “*tentative*,” and “*informative*” based on three areas that are the area of *refusal, irresolution*, and *acceptance*, respectively.
7. Repeat the procedure until all features are either characterized as “*non-informative*” or “*informative*” or the algorithm has reached the predefined limit of the random forest runs.

#### Model Building

In this section, we elected to model the relationship between the SYNTAX score response and the set of predictors based on the utilization of a two-part model inspired by a well-known class of statistical models, namely the *Hurdle* models (16). The Hurdle models are used for data having a large number of zeros as one component, and a distribution of non-zero values as second component. Hence, these models attempt to capture both the absence (or presence) of a binary response (hurdle component) and the magnitude of the non-zero outcome.

Describing briefly, in our experimental setup, the *Model Building* phase consists of a two-step process that deploys (*i*) a classification step, modelling a binary response, which discriminates patients into two groups, i.e., patients with a zero SYNTAX score (*Y* = 0) and patients with non-zero, positive SYNTAX score (*Y* = 1), and (*ii*) a regression step, modelling the SYNTAX score for patients with non-zero SYNTAX score (*Y* > 0). Following the terminology used for the Hurdle models, the former is referred as the *zero-part* model and the latter is referred as the *truncated count-part* model (Figure 1), which practically means that if a patient presents with a zero SYNTAX score, the threshold to the truncated count part is not crossed, and a zero value for SYNTAX score is assigned to this patient. Otherwise, the threshold to the truncated count part is crossed, and a SYNTAX score above zero is observed. In the latter case, the count-part model is triggered, providing, in turn, the expected positive value of the actual SYNTAX score.

Given the fact that there is a plethora of classification and regression candidates that can be used for building the zero- and count-part models, we decided to investigate a specific set of well-established statistical and ML algorithms that have been extensively applied in other experimental studies. More specifically, we made use of four classifiers and their counterpart regression techniques that are summarized in Table 1 along with a brief description of their basic principles.

**Table 1:** Classification and regression methods for building the zero- and count-part models.

**Method**	**General idea**
Regression Analysis (RA) variant with Logistic Regression and Linear Regression for fitting the zero- and count-part models, respectively.	Logistic Regression employs a logit function for estimating the log odds of a binary response and probabilities for differentiating the cases into negative (absence)/positive (presence) classes.
	Linear Regression estimates the parameters (regression coefficients) of a known explicit linear function describing the relationship between a continuous response and a set of predictors minimizing the sum of square residuals.
Classification and Regression Tree (CART) for fitting the zero- and count-part models.	Build hierarchical models composed of decision nodes and leaves to predict the class (or continuous outcome) of a response based on a set of predictors.
Random Forest (RF) for fitting the zero- and count-part models.	An ensemble algorithm that combines a set of votes (or continuous outcomes) evaluated by a set of individual decision trees estimations.
Support Vector (SV) variant with Support Vector Machines (SVM) and Support Vector Regression (SVR) for fitting the zero- and count-part models, respectively.	SVM finds the optimal hyperplane separating the cases into negative (absence)/positive (presence) classes margin between the data points to classify them into predefined classes.
	SVR is an extension of SVM sharing the same principles but with the aim of estimating a continuous outcome for a response variable.

The building process of the candidate models was based on a *leave-one-out cross-validation* (LOOCV) data-generating schema partitioning the available dataset into *training* and *test* sets. The training sets were used as the basis for fitting each candidate model, whereas the test sets were then used for evaluating the predictive power of each model. Regarding the performance evaluation, a variety of well-known measures for both classification and regression tasks have been computed for assessing the quality of the set of competing ML models. In particular, for the zero-part model, we made use of measures (Table 2) derived from the *confusion matrix*, indicating the instances of each class [positive (+) and negative (-)] that are either correctly [*True Positives* (*TP*) and *True Negatives* (*TN*)] or erroneously classified [*False Positives* (*FP*) and *False Negatives* (*FN*)]. On the other hand, the evaluation of the prediction performance for the regression models was based on specific *loss functions* *l*(*y*_*i*_, ŷ_*i*_), which compute the discrepancy between the *actual* *y*_*i*_ and the *estimated* (or *predicted*) ŷ_*i*_ values for each instance of the test set.

**Table 2:** Performance evaluation metrics for classification and regression tasks.

**Task**	**Measure**	**Definition**
Classification (zero-part model)	Accuracy	$Accuracy=\frac{TP+TN}{TP+FP+TN+FN\;}$
	Balanced accuracy	$Balanced\;Accuracy=\frac{sensitivity+specificity}{2\;}$
	Precision	$Precision=\frac{TP}{TP+FP}$-
	Recall	$Recall=\frac{TP}{TP+FN}$
	F-measure	$F-measure=\frac{{\left(1+\beta \right)}^{2}•Recall•Precision}{\beta^{2}•Recall•Precision}$
Regression (count-part model)	Median Error (MdE)	*median*{*E*_*i*_}, where *E*_*i*_ = (*y*_*i*_ − ŷ_*i*_)
	Median Absolute Error (MdAE)	*median*{*AE*_*i*_}, where *AE*_*i*_ = |*y*_*i*_ − ŷ_*i*_|
	Median Magnitude of Relative Error (MdMRE)	*median*{*MRE*_*i*_}, where $MRE_{i}=|\frac{y_{i}-{ŷ}_{i}}{y_{i}}|•100$
	Median Magnitude of Relative Error to the Estimate (MdMER)	*median*{*MER*_*i*_}, where $MER_{i}=|\frac{y_{i}-{ŷ}_{i}}{{ŷ}_{i}}|•100$

The proposed framework was implemented using the open-source statistical programming language R (17). In all tests, a difference was considered statistically significant when the *p*-value (significance) was < 0.05 (*p* ≤ 0.05). All the tests conducted were two-tailed (non-directional) in the sense that the alternative hypothesis is that the measures tested are not equal.

## Results

**Figure 2:**
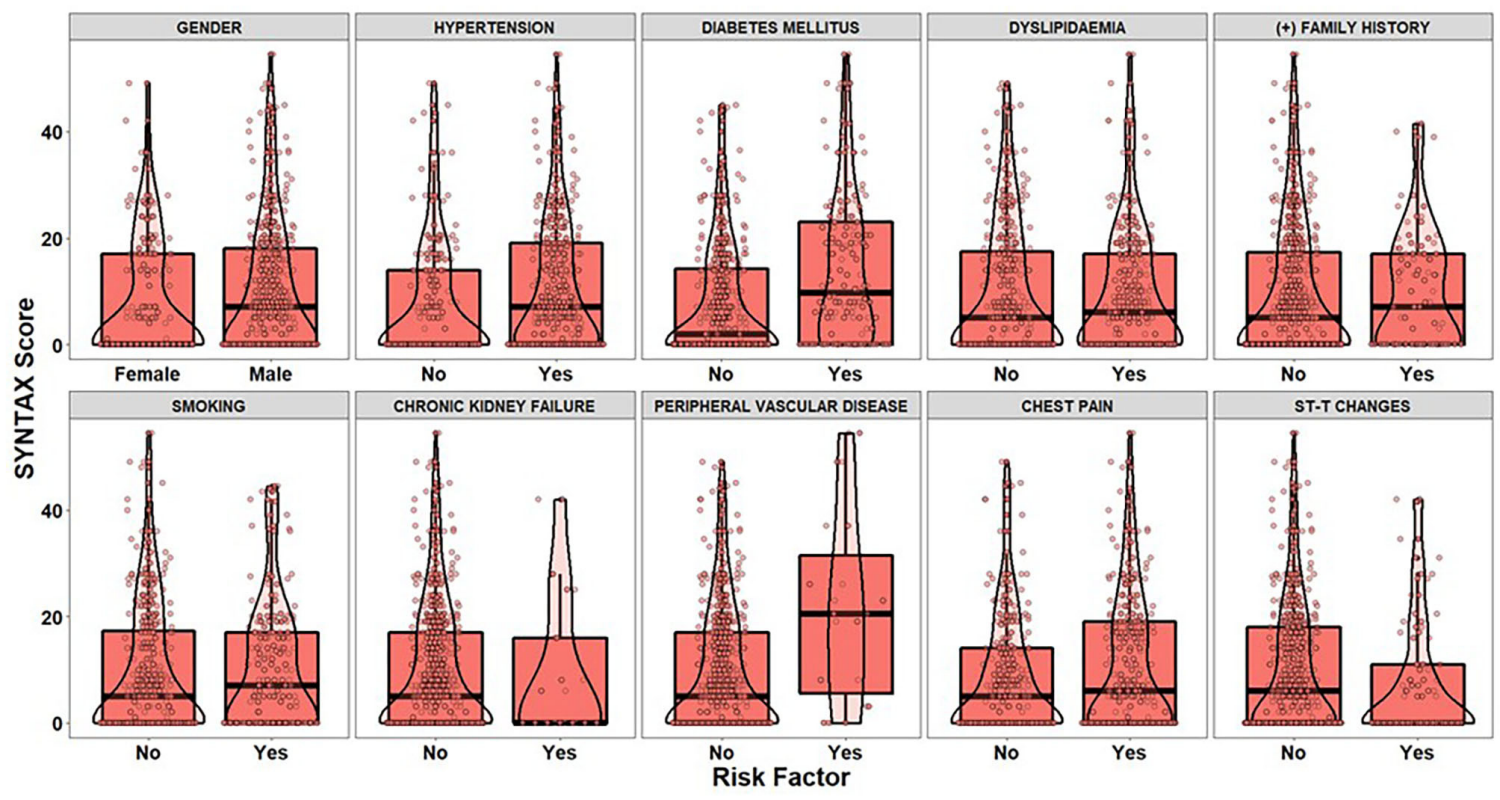
The boxplots and violin plots represent the distributions of the SYNTAX score of patients (dots) for each level of categorical risk factor.

After applying the inclusion and exclusion criteria for this analysis, our sample size finally included 303 patients with suspected CAD. Tables 3, 4 summarize the findings from the descriptive statistics analysis concerning the set of predictors indicated as potential risk factors that may affect the distribution of SYNTAX score. In addition, exploratory analysis along with appropriate statistical hypothesis procedures were also conducted with the aim of investigating patterns between the response (SYNTAX score) and the set of risk factors. Regarding the statistical hypothesis tests (Table 3), the non-parametric *Mann-Whitney U test* was used for examining, whether there was a statistically significant effect of categorical risk factors on the distribution of the SYNTAX score response, since SYNTAX score distribution was highly skewed with an excess of zero values (Figure 2). This was also the reason for the utilization of the non-parametric *Spearman's rho coefficient* for investigating statistically significant correlations between the SYNTAX score and the set of continuous risk factors (Table 4).

**Table 3:** Descriptive and exploratory analyses for categorical risk factors and SYNTAX score.

			**SYNTAX score**		
**Risk factor**	**Group**	***N* (%)**	***M* (*SD*)**	***Mdn* [*min, max*]**	***p***
Gender	Female	90 (29.70)	8.29 (11.46)	0.00 [0, 49.0]	0.061
	Male	213 (70.30)	10.79 (12.78)	7.00 [0, 54.5]	
Hypertension	No	109 (35.97)	8.45 (12.00)	0.00 [0, 49.0]	**0.025**
	Yes	194 (64.03)	10.94 (12.62)	7.00 [0, 54.5]	
Diabetes mellitus	No	215 (70.96)	8.29 (11.18)	2.00 [0, 45.0]	**<0.001**
	Yes	88 (22.04)	14.34 (14.27)	9.75 [0, 54.5]	
Dyslipidaemia	No	163 (53.80)	10.04 (12.64)	5.00 [0, 49.0]	0.757
	Yes	140 (46.20)	10.05 (12.24)	6.00 [0, 54.5]	
Positive (+) family history of CAD	No	252 (83.17)	10.00 (12.64)	5.00 [0, 54.5]	0.705
	Yes	51 (16.83)	10.29 (11.51)	7.00 [0, 41.5]	
Smoking	No	196 (64.69)	9.71 (12.55)	5.00 [0, 54.5]	0.353
	Yes	107 (35.31)	10.66 (12.25)	7.00 [0, 44.5]	
Chronic kidney failure	No	290 (95.71)	10.07 (12.39)	5.00 [0, 54.5]	0.651
	Yes	13 (4.29)	9.62 (13.93)	0.00 [0, 42.0]	
Peripheral vascular disease	No	292 (96.37)	9.60 (11.95)	5.00 [0, 49.0]	**0.014**
	Yes	11 (3.63)	21.82 (18.88)	20.50 [0, 54.5]	
ST-T changes	No	252 (83.17)	10.48 (12.55)	6.00 [0, 54.5]	0.088
	Yes	51 (16.83)	7.89 (11.74)	0.00 [0, 42.0]	

*Risk factors presenting a statistically significant effect on SYNTAX score are highlighted in bold font*.

**Table 4:** Descriptive and exploratory analyses for continuous risk factors and SYNTAX score.

**Risk factor**	***M***	***SD***	***Mdn***	***Min***	***Max***	***Spearman rho* (*p*)**
Age (in years)	64.25	11.13	66.00	24.00	87.00	0.092 (0.110)
Body mass index (BMI) (kg/m^2^)	28.85	4.97	28.40	12.20	44.30	−0.083 (0.151)
Systolic arterial pressure (SAP) (mmHg)	136.08	17.85	135.00	85.00	200.00	**0.120 (0.036)**
Diastolic arterial pressure (DAP) (mmHg)	80.17	9.62	80.00	49.00	110.00	−0.017 (0.767)
Glomerular filtration rate (GFR) by CKD-EPI (mL/min/1.73m2)	93.15	34.43	89.00	6.10	254.40	−0.089 (0.121)
UREA (mg/dL)	40.97	20.58	36.00	0.84	177.00	0.062 (0.285)
Total cholesterol (CHOL) (mg/dL)	164.73	42.14	162.00	5.50	341.00	−0.045 (0.438)
High density lipoprotein cholesterol (HDL) (mg/dL)	45.47	14.80	43.00	18.00	109.00	**−0.149 (0.009)**
Aspartate aminotransferase (SGOT) (units/L)	21.38	11.20	19.00	4.00	102.00	−0.032 (0.578)
Alanine aminotransferase (SGPT) (units/L)	23.25	14.76	19.00	3.00	114.00	−0.013 (0.819)
Hemoglobin (HGB) (g/dL)	14.02	1.65	14.00	4.52	18.80	−0.053 (0.354)
Platelets (PLT) (*1000)	232.38	65.02	227.00	70.00	599.00	0.065 (0.262)
White blood cells (WBC) (*1000)	7.51	1.98	7.31	1.06	14.90	**0.142 (0.013)**
$Monocyte-to-HDL-cholesterol\;ratio\;\left(RATIO1\right)=\frac{\left(MONO\%\times WBC\right)}{HDL}$	1.51	0.81	1.41	0.04	6.77	**0.118 (0.040)**
*Lymphocyte* − *to* − *monocyte ratio* (*RATIO*2)	0.33	0.17	0.29	0.01	1.47	0.013 (0.826)
Atherogenic index of plasma levels ($RATIO3=\log\left(\frac{TG}{HDL}\right)$)	0.47	0.32	0.46	−0.21	1.73	**0.180 (0.002)**

*Statistically significant correlations are highlighted in bold font*.

The visual examination through the construction of boxplots and violin plots for the SYNTAX score (Figure 2) and each level of the categorical risk factors demonstrates substantial differences between the distributions for a subset of risk factors. More specifically, patients with Hypertension, Diabetes Mellitus and Peripheral Vascular Disease have generally higher values of SYNTAX score compared to patients without that medical history. Indeed, the non-parametric Mann-Whitney test indicated statistically significant effects of Hypertension (*p* < 0.025), Diabetes Mellitus (*p* < 0.001), and Peripheral Vascular Disease (*p* = 0.014) on the SYNTAX score distribution (last column of Table 3). As far as the continuous risk factors are concerned, the non-parametric *Spearman's rho* coefficient revealed a statistically significant negative correlation between high-density lipoprotein (HDL) levels and SYNTAX score [*r*(303) = −0.149, *p* = 0.009] and positive correlations between systolic arterial pressure (SAP) [*r*(303) = 0.120, *p* = 0.036], white blood cells (WBC) [*r*(303) = 0.142, *p* = 0.013], Monocyte-to-HDL ratio [*r*(303) = 0.118, *p* = 0.040] and Atherogenic Index of Plasma (AIP) levels [*r*(303) = 0.180, *p* = 0.002] and the SYNTAX score response.

To conclude, the findings derived from the exploratory analysis and hypothesis testing procedures showcased that there is a set of risk factors, which can be considered as important candidate predictors for estimating the SYNTAX score of a patient. On the other hand, the analysis provides empirical evidence for risk factors affecting the whole distribution of SYNTAX score and not specific information regarding the risk factors that should participate into the building of the two-part model (zero-part and count-part). In other words, there is a lack of knowledge concerning (a) the mechanism discriminating the patients into zero/non-zero groups and (b) the mechanism that efficiently models the relationship between positive values of SYNTAX score and the subset of predictors. The latter can be considered as a challenge of practical importance, since the development of an accurate clinical risk-score algorithm that leads, in turn, to an effective personalized patient management should be based on techniques able to model the underlying relationships for both zero-part and count-part models.

**Figure 3:**
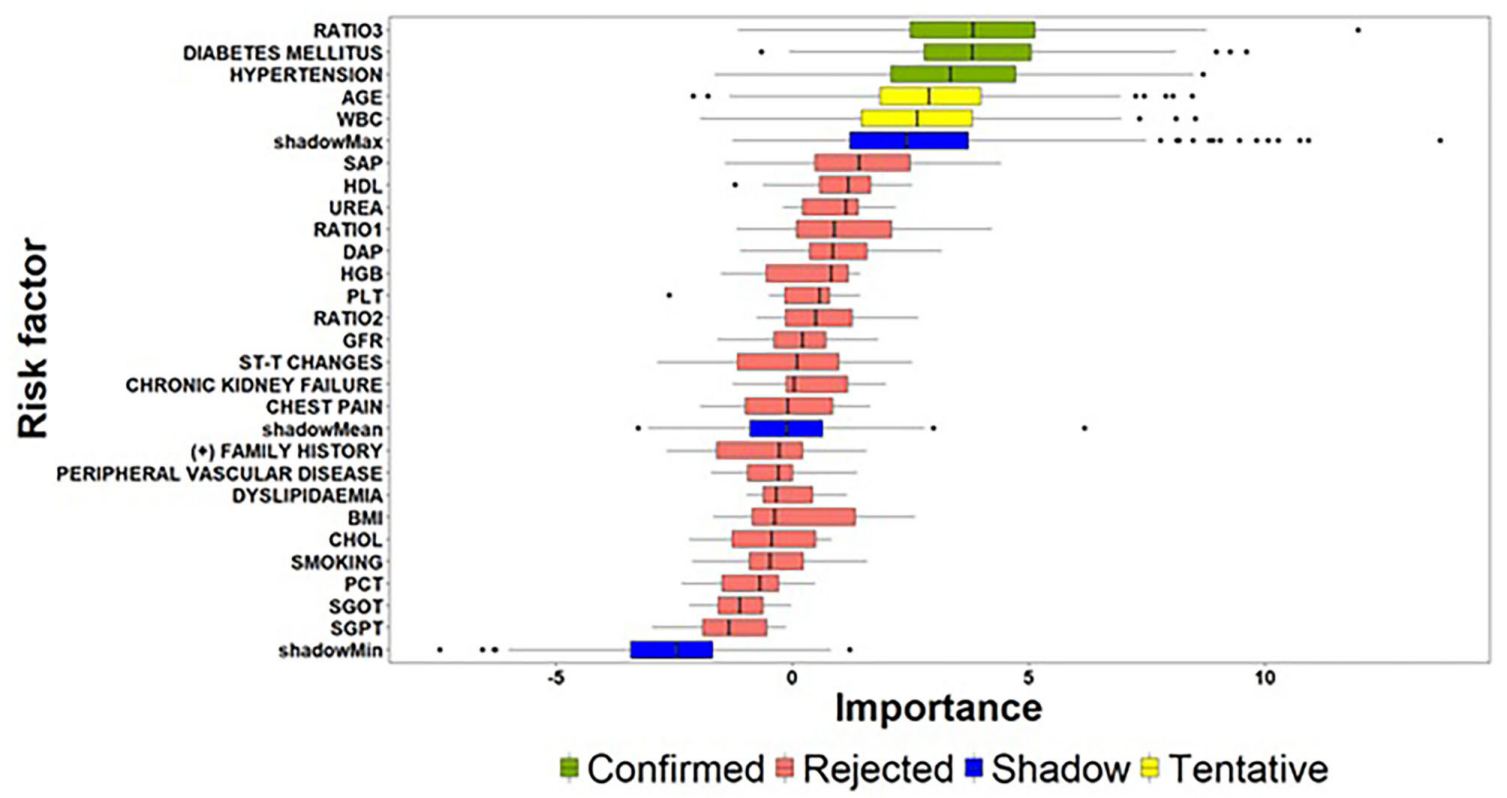
Importance of features extracted by the Boruta algorithm (zero-part) (the abbreviations of the risk-factors can be found in Table 4) [Ratio 1: *Monocyte* − *to* − *HDL* − *cholesterol ratio*; Ratio 2: *Lymphocyte* − *to* − *monocyte ratio*; Ratio 3: *Atherogenic Index of Plasma levels* ($\log\left(\frac{TG}{HDL}\right)$].

To alleviate the challenging task of identifying the critical risk factors affecting the dependent variables in both the zero- and count-part components, the Boruta algorithm was deployed twice for modelling the underlying relationships. Figure 3 visualizes the results derived from the execution of the Boruta algorithm on the building phase of the zero-part model. In particular, the boxplots visualize the distributions of the *z*-scores produced by the Boruta algorithm for the examined risk factors, where risk factors confirmed as “*informative*” are highlighted with green colour. In contrast, risk factors that were characterized as “*non-informative*” are depicted with purple colour. Furthermore, risk factors yielding important scores close to the best shadow attribute (blue colour) are characterized as “*tentative*” (yellow colour), and an additional step was conducted for deciding whether a tentative attribute should be finally confirmed as informative or non-informative based on the comparison of its median *z*-score with the median *z*-score of the best shadow attribute.

Considering the above guidelines related to the interpretation of Figure 3, the results of the Boruta algorithm for the zero-part model suggest a subset of three significant risk factors affecting the discrimination of patients into zero and non-zero groups. Atherogenic Index of Plasma levels (Ratio 3) and Diabetes Mellitus followed by Hypertension can be considered as the most important risk factors for the zero-part model since they present very close median values and similar distributions. An interesting conclusion derived from the analysis is the fact that beside these three risk factors, Age and WBC are characterized as tentative by the algorithm. This practically means that Age and WBC may present significant effects on discriminating patients into zero and non-zero SYNTAX score groups and there is a need for further experimentation, when new patient records will be available.

**Figure 4:**
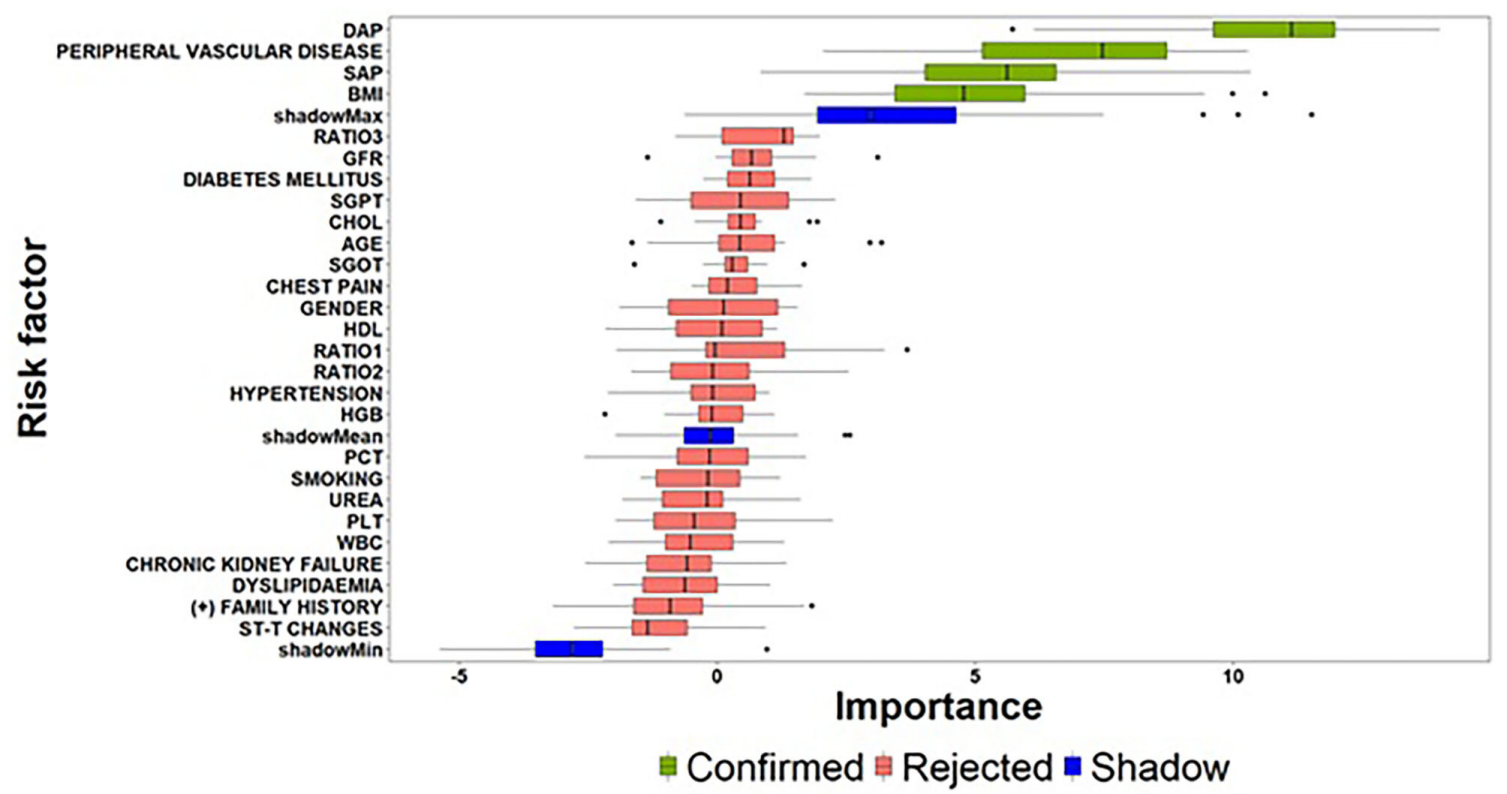
Importance of features extracted by the Boruta algorithm (count-part) (the abbreviations of the risk-factors can be found in Table 4).

As far as the count-part model concerns, Figure 4 summarizes the risk factors with significant effects on the strictly positive distribution of the SYNTAX score response. Among the examined risk factors, DAP, Peripheral Vascular Disease, BMI and SAP were identified as important features for providing an accurate estimate of the actual value of SYNTAX score given that a patient presented with a non-zero SYNTAX score. Additionally, among the four identified important risk factors, DAP exhibited a considerably high *z*-value compared to the other risk factors indicating a significant contribution to the accurate prediction of the SYNTAX score. At this point, we have also to note that the Boruta algorithm did not result into tentative variables, which practically means that the identified solution provides strong empirical evidence for the set of risk factors affecting the strictly positive distribution of SYNTAX score.

Summarizing the results related to the former pillar of this study, that is the investigation of important risk factors affecting the zero- and count-part models, a first interesting finding concerns the identification of different sets of clinical risk factors associated to the two-step modelling technique. On the one hand, the task of predicting, whether a patient is at risk for presenting with a non-zero SYNTAX score is of great importance, since the clinical management will be focused to greater awareness even before hospitalization and the procedure of coronary angiography. Similarly, a patient predicted with zero SYNTAX score will avoid coronary angiography and get more accurately counselling to follow lifestyle instructions and periodical medical examination. To this regard, a set of three risk factors (Atherogenic Index of Plasma levels, Diabetes Mellitus and Hypertension) was identified as critical for deciding, whether a patient is at risk for presenting a non-zero SYNTAX score. Based on this decision, the next crucial question concerns the estimation of the expected SYNTAX score given that a patient is classified into the non-zero group since the accurate evaluation of SYNTAX score will lead, in turn, to different personalized patient management based on the severity of CAD determined by the estimated SYNTAX score. The findings derived from the proposed feature selection algorithm suggest that a set of different risk factors (DAP, Peripheral Vascular Disease, BMI, and SAP) are responsible for the changes presented in the strictly positive SYNTAX score distribution.

Moreover, the experimentation on the available medical and clinical records of patients revealed that the process of building an accurate prediction algorithm should take into consideration the different piece of information related to the two-part model. Although the exploratory analysis and the statistical hypothesis procedures provide meaningful guidance for the examination of potential risk factors affecting the SYNTAX score response, they are essential for extracting knowledge concerning the whole distribution of SYNTAX score. Indeed, the aggregated findings derived from the consecutive execution of the Boruta algorithm on the zero- and count-part models are quite similar to the results extracted from the conduction of the exploratory analysis and hypothesis testing procedures but on the same time, they also present few divergences. More specifically, based on the aggregated set of risk factors (both zero- and count-part models), five out of a total of seven risk factors are identified as significant by both the proposed feature selection mechanism and the traditional hypothesis test procedures. On the other hand, the adopted feature selection algorithm can be considered as a more dedicated approach providing straightforward guidance fulfilling better insights about the different mechanisms that are responsible for the two-part modelling process rather than extracting meaningful information for the whole distribution of SYNTAX score. Moreover, the Boruta algorithm resulted to the identification of two additional risk factors (DAP and BMI) related to the count-part model, which were not reported as statistically significant through the evaluation of the Spearman's correlation coefficient. A possible explanation for this inconsistency may lie on the fact that the non-parametric correlation coefficient is not able to detect the relationship between the response and these specific factors, due to the presence of excess zeros in the distribution of SYNTAX score. Indeed, the evaluation of the non-parametric Spearman correlation coefficient for the strictly positive SYNTAX scores indicated a statistically significant negative correlation for both DAP [*r*(173) = −0.225, *p* = 0.003] and BMI [*r*(173) = −0.172, *p* = 0.024] risk factors.

After the identification of the significant risk factors, we focused on the performance evaluation for the set of examined classifiers (Table 5) and regression models (Table 6) that are related to the zero- and count-part models, respectively. Table 5 summarizes the performance metrics for the zero-part models trained and test through LOOCV validation scheme. The findings indicate the superiority of two specific classifiers in terms of prediction capabilities as measured by different metrics. More specifically, LR presents consistently the best performance in three out of five measures (*Accuracy, Balanced Accuracy, Precision*). In contrast, Support Vector Machines (SVM) classifier seems to perform best in terms of both *Recall* and *F*_1_ measure. As far as the count-part model concerns, Table 6 suggests the dominance of Support Vector Regression (SVR) model for evaluating the SYNTAX score of a patient given that he/she presents with a non-zero SYNTAX score.

**Table 5:** Performance evaluation results of zero-part models (classification task).

**Model**	**CART**		**LR**		**RF**		**SVM**	
**Measure**	**Training**	**Test**	**Training**	**Test**	**Training**	**Test**	**Training**	**Test**
Accuracy	**0.6964**	0.5545	0.6304	**0.6106**	0.6436	**0.6106**	0.5941	0.5809
Balanced accuracy	**0.6843**	0.5391	0.6075	**0.5892**	0.6085	0.5748	0.5394	0.5240
Precision	**0.7189**	0.6022	0.6488	**0.6368**	0.6407	0.6190	0.5926	0.5839
Recall	0.7688	0.6474	0.7679	0.7399	0.8555	0.8266	**0.9249**	**0.9249**
F1	**0.7430**	0.6240	0.7037	0.6845	0.7327	0.7079	0.7223	**0.7149**

*The best classifier in terms of each performance measure is denoted in bold font for both training and test sets*.

**Table 6:** Performance evaluation results of count-part models (regression task).

**Model**	**CART**		**LR**		**RF**		**SVR**	
**Measure**	**Training**	**Test**	**Training**	**Test**	**Training**	**Test**	**Training**	**Test**
MdE	−1.3125	−1.3548	−2.3666	−2.4494	−1.5827	−1.8972	**−0.4284**	**−0.4383**
MdAE	6.3125	8.5806	7.5393	7.7431	**5.6916**	7.5513	6.3216	**6.5032**
MdMRE	0.3567	0.4885	0.4511	0.4679	**0.3388**	0.4451	0.4340	**0.4368**
MdMER	**0.3514**	**0.4440**	0.4469	0.4624	0.3715	0.4604	0.4352	0.4535

*The best regression model in terms of each performance measure is denoted in bold font for both training and test sets*.

The final phase of the framework concerns the deployment of the proposed data-driven ML approach in real life environments. To this regard, a critical issue is to realize, whether the extracted solution is both interpretable and understandable from a clinical practitioner's point of view. Indeed, the practical evidence suggests that Hypertension and Diabetes Mellitus are amongst the major risk factors for the development and progression of CVDs (18). Similarly, SBP and DBP as well as the Atherogenic Index of Plasma belong to traditional risk indicators associated with the occurrence and development of CVDs (19–22). Also, AIP is proposed as a strong biomarker for predicting the risk of cardiovascular events in patients with Hypertension, Metabolic Syndrome and Diabetes Mellitus (23, 24). Similarly, it has also been shown that BMI represents a significant prognostic indicator for various CVDs (25). Peripheral Vascular Disease comorbidity has been also linked with higher prevalence of major adverse cardiovascular events among CAD patients (26, 27).

## Discussion and Future Work

The massive load of the molecular data generated from several research efforts need the development of data analytics and ML algorithms for extracting knowledge hidden in data and for synthesizing the body of knowledge. Such necessity is further stressed in CVDs from the heterogenous nature of the data that must be in depth explored to allow the practical utility of clinically relevant information, since they are derived from various sources, e.g., clinical, molecular, chemical, and epidemiological. In recent years, the rapid increase in computational power allows data-driven analytical solutions to be applied on diverse dataset collections to generate predictive models of pattern associations of prognostic, diagnostic and therapeutic value (3–5). To this end, the capacity for the efficient clinical translation of the existed risk factor variables in CVDs coincides with the development of predictive models to accurately achieve the clinical validation of the associated outcome in patient populations and/or individuals (28, 29). By developing ML solutions as hands-on approaches to precisely predict in-real time the disease prognosis, this direction empowers robust personalized medicine decisions for specific populations, or individual patients, in the clinical setting. Previous efforts aiming to advance the application of ML prediction models in CVDs have been previously executed in patients suffering from ST-elevated myocardial infarction (STEMI) (30) and non-ST-segment elevation myocardial infarction (non-STEMI) (31).

It is of crucial clinical significance the capacity of developing powerful risk-stratification algorithms (pre-test probability testing models) with the aim of predicting the risk of severe CAD in patients presenting with suspected symptoms. Such efforts are in concordance with the current healthcare environment where the power in computerized systems allows the clinical implementation of data-driven ML platforms to guide therapeutic decisions in the clinical setting. To this end, the handling and monitoring of CVD patients have been tremendously benefited from such artificial intelligence-based algorithms and practices (32, 33). Importantly, the use of ML frameworks provides to interfaces the needed automate connection of information coming from various sources and facilitates practitioners to improve clinical outcomes in their practice. Consequently, the continuous application of ML algorithms clearly contributes to the progression of precision cardiovascular medicine. As far as CAD is concerned, the implementation of ML algorithm approaches has been applied in the clinical setting among others (*i*) to predict the occurrence of obstructive CAD by evaluating various clinical variables and the coronary artery calcium score (34); (*ii*) to improve the detection of functionally obstructive CAD (35), as well as to detect lesion-specific ischemia (36) by using computational fluid dynamics algorithms; (*iii*) to estimate the pre-test probability of CAD (37); and (*iv*) to evaluate the automatic prediction of obstructive CAD from myocardial perfusion imaging (38, 39).

The present clinical status for patients suspected with CAD relies on invasive coronary angiography and the estimation of the SYNTAX score afterwards for their management and therapeutic approach. Since all these patients are subjected to this invasive procedure, it is stressfully demandable to develop predictive risk-stratification models to precisely identify individuals with zero SYNTAX score and thus avoiding such handling. In this study, we proposed a data-driven approach with the aim of identifying a set of critical risk factors that affect the SYNTAX score of patients with suspected CAD. More specifically, we proposed a two-step technique for modelling the underlying relationships between the set of important risk factors and SYNTAX score that evaluates the complexity of CAD by separating the process into (a) a binary classification task for discriminating patients into two groups (zero/non-zero SYNTAX score) and (b) a regression task for providing an expectation of the actual SYNTAX score given the fact that a patient belongs to the non-zero SYNTAX score group. The reason behind this choice was the difficulties arisen in the modelling process of SYNTAX score response through traditional statistical methods due to the existence of an excessive number of patients presenting with a zero SYNTAX score and the overdispersion of the corresponding distribution. Moreover, the proposed methodology inspired by the Hurdle models, provides a straightforward mechanism for understanding how different predictors are associated to a highly skewed response with an excess of zero values, since it combines one process for zero counts and another process for strictly positive counts. To this regard, the framework makes use of an efficient feature selection algorithm that extracts a set of potential different risk factors for the two-step model.

The results from the deployment of the proposed approach indicated that there is, indeed, an imperative need for the development of efficient data-driven ML frameworks, since the accurate prediction of SYNTAX score is a complicated task, in which different risk factors contribute to its scalable values be actually reached in each patient with CAD. The developed risk-stratification ML framework aims to facilitate the clinicians to identify which of the patients presenting with suspected CAD should be referred for further functional or anatomical diagnostic testing and which of them should undergo emergency coronary angiography and/or a coronary revascularization procedure.

In terms of the count-part analysis, our model reflects a growing effort for a bloodless coronary angiography in cardiology, with the potential to assess patients with SYNTAX score > 0. An observational study (6) in a large cohort of 398,978 patients without known CAD undergoing elective cardiac catheterization yielded that more than one third of them had complex obstructive CAD. This renders imperative the need to identify soon enough which of the presenting patients may warrant urgent revascularization procedure to be performed. On the contrary, a recent report (40) suggests that at least half individuals undergoing invasive coronary angiography do not earn some benefit from it, and that the traditional use of obstructive stenosis on coronary angiography might no longer be an adequate definition of CAD. Thus, functional tests and the development of risk-score models, such as our count-part model, which can quantify bloodlessly the extent of CAD, could be useful to initially approach the severity and complexity of a patient's CAD and guide further therapeutic approaches.

Undoubtedly, however, our study is subject to limitations. Firstly, our model was not compared in terms of performance with other widely used pre-test clinical scores for the assessment of severe CAD. Most importantly, the restricted sample size of our study limits the generalizability of our findings and the created zero- and count-part models should be also externally validated in larger CAD populations, in an effort to broadly contribute to a more personalized patient management and counselling within the healthcare environment.

An interesting direction for future work concerns the deployment of the framework and its practical evaluation, when more data will be available. Moreover, in this study, we opted to base the building process of the two-step model on a specific set of ML algorithms. Certainly, there is a plethora of alternative ML methods that can be used for fitting both the zero- and count-part models that deserves extensive experimentation for implementation and clinical practical utility. Another dynamic topic for further research that it is still challenging in this era deals with the enrichment of the proposed framework with genotype specific information covering clinically validated biomarkers extracted through bioinformatic and pharmacogenomic analysis. Indeed, as it has already been published by our research group, the GESS trial, for each patient enrolled in the study, incorporates the genotyping analysis of 228 SNP biomarkers that cover 127 genes being affected by the presence of one or more SNPs (13). Such approach is complementary to the work presented above since it will allow us to clinically evaluate the practical utility of this panel of SNP biomarkers in predicting CAD severity. By achieving the latter task, the statistically determined relevant panel of SNPs that provides the predictive capacity, it will be incorporated as genetic score into the algorithms of the risk-stratification ML framework to enhance its accuracy and strength. In this regard, the proposed genetic implementation in the developed ML risk stratification model is quite challenging, since it will allow clinicians to easily adopt predictive risk score variables for the severity of CAD in their practice by using in real-time clinical, laboratory and genetic parameters to stratify and accordingly clinically handle individual patients. To this end, our research team is working towards the development of an open-source web platform augmented with an interactive dashboard that will facilitate the adoption and extension of the proposed ML framework targeting at different stakeholders that are clinical practitioners, health care managers, researchers and, generally, society.

In conclusion, once the created zero- and count-part models get externally validated, they could help clinicians to identify which of the patients presenting with suspected CAD should be just referred for further non-invasive diagnostic testing and which of them should urgently undergo coronary angiography and/or a coronary revascularization procedure. This would ultimately ease clinical decision-making process, patient management and counselling.

## Data Availability Statement

The original contributions presented in the study are included in the article/Supplementary Material, further inquiries can be directed to the corresponding author/s.

## Ethics Statement

The studies involving human participants were reviewed and approved by the Scientific Committee of AHEPA University Hospital (reference number 12/13-06-2019). The patients/participants provided their written informed consent to participate in this study.

## Author Contributions

NM, LA, FC, and IV: conceptualization and ML model development. GSo, EK, NS, DM, ASP, and GSi: patient recruitment, sample collection, and clinical evaluation. LA and IV: supervision of the study. All authors contributed to ML model validation, data interpretation, writing of the manuscript, read, and approved the final version of the manuscript.

## Funding

This research has been co-financed by the European Regional Development Fund of the European Union and Greek National Funds through the Operational Programme Competitiveness, Entrepreneurship, and Innovation, under the call RESEARCH–CREATE–INNOVATE (project code: T1EDK-02354).

## Conflict of Interest

FC is employed by Labnet Laboratories. The remaining authors declare that the research was conducted in the absence of any commercial or financial relationships that could be construed as a potential conflict of interest.

## Publisher's Note

All claims expressed in this article are solely those of the authors and do not necessarily represent those of their affiliated organizations, or those of the publisher, the editors and the reviewers. Any product that may be evaluated in this article, or claim that may be made by its manufacturer, is not guaranteed or endorsed by the publisher.
