# Supplementary material for: A Risk-Stratification Machine Learning Framework for the Prediction of Coronary Artery Disease Severity: Insights From the GESS Trial
Source: Front Cardiovasc Med. 2022 Jan 18;8:812182. doi: 10.3389/fcvm.2021.812182 (PMC8804295; doi:10.3389/fcvm.2021.812182)
Supplement: Supplementary file 1 [file Data_Sheet_1.DOCX]

**Supplementary Table. Study population collected clinical variables and measured parameters**

| **Electronic Health Record** | **Variable Name** | **Description** | **Type** | **Categories** |
| --- | --- | --- | --- | --- |
| HISTORY | ID |  | Numeric |  |
|  | GENDER | Gender | Categorical | Female/Male |
|  | HYPERTENSION | History of hypertension | Categorical | No/Yes |
|  | DIABETES MELLITUS | History of diabetes mellitus | Categorical | No/Yes |
|  | DYSLIPIDAEMIA | History of dyslipidaemia | Categorical | No/Yes |
|  | (+) FAMILY HISTORY | Positive (+) family history of CAD | Categorical | No/Yes |
|  | SMOKING | History of smoking | Categorical | No/Yes |
|  | AGE | Age of patient (in years) | Numeric |  |
|  | PREVIOUS STROKE | Previous stroke | Categorical | No/Yes |
|  | CHRONIC KIDNEY FAILURE | History of chronic kidney disease | Categorical | No/Yes |
|  | PERIPHERAL VASCULAR DISEASE | History of peripheral vascular disease | Categorical | No/Yes |
|  | AORTIC ANEURYSMS | History of aortic aneurysms | Categorical | No/Yes |
|  | CHRONIC PULMONARY OBSTRUCTIVE DISEASE | History of chronic pulmonary obstructive disease | Categorical | No/Yes |
|  | AUTOIMMUNE DISEASE | History of any autoimmune disease | Categorical | No/Yes |
|  | ATRIAL FIBRILLATION | History of atrial fibrillation | Categorical | No/Yes |
| DIFFERENTIAL | ACS | Acute coronary syndrome | Categorical | No/Yes |
|  | NSTEMI | Non-ST-elevated myocardial infarction | Categorical | No/Yes |
|  | STEMI | ST-elevated myocardial infraction | Categorical | No/Yes |
|  | UNSTABLE ANGINA | Unstable angina | Categorical | No/Yes |
|  | STABLE ANGINA | Stable angina | Categorical | No/Yes |
|  | SPECT | Pathological single-photon emission computerized tomography results | Categorical | No/Yes |
|  | CCTA | Pathological coronary computed tomography angiography results | Categorical | No/Yes |
|  | THORACIC PAIN | Thoracic pain | Categorical | No/Yes |
|  | CHRONIC CORONARY SYNDROME | Chronic coronary syndrome | Categorical | No/Yes |
|  | SEVERE AORTIC STENOSIS | Severe aortic stenosis | Categorical | No/Yes |
|  | HEART FAILURE | Heart failure | Categorical | No/Yes |
| ENTRY | CHEST PAIN | Chest pain | Categorical | No/Yes |
|  | DYSPNEA | Dyspnea | Categorical | No/Yes |
|  | EASY FATIGUE | Easy fatigue | Categorical | No/Yes |
|  | BMI | Body mass index (kg/m^2^) | Numeric |  |
|  | BPM | Beats per minute (heart rate) | Numeric |  |
|  | SAP | Systolic arterial pressure (SAP) (mmHg) | Numeric |  |
|  | DAP | Diastolic arterial pressure (DAP) (mmHg) | Numeric |  |
|  | CRUSADE SCORE | Crusade score | Categorical | No/Yes |
|  | GRACE SCORE | Grace score | Categorical | No/Yes |
|  | QRS DURATION (ms) | QRS duration (in ms) | Numeric | No/Yes |
|  | ST-T CHANGES | ST-T changes | Categorical | No/Yes |
| BIOCHEMICAL | GFR | Glomerular filtration rate by CKD-EPI (mL/min/1.73m2) | Numeric |  |
|  | GLU | Glucose (mg/dL) | Numeric |  |
|  | UREA | Urea (mg/dL) | Numeric |  |
|  | CREATININE | Creatinine (mg/dL) | Numeric |  |
|  | URIC ACID | Uric acid (mg/dL) | Numeric |  |
|  | CHOL | Total Cholesterol (mg/dL) | Numeric |  |
|  | TG | Triglycerides (mg/dL) | Numeric |  |
|  | HDL | High density lipoprotein cholesterol (mg/dL) | Numeric |  |
|  | LDL | Low density lipoprotein cholesterol (mg/dL) | Numeric |  |
|  | TNT-HS | High sensitivity cardiac troponin (ng/L) | Numeric |  |
|  | SGOT | Aspartate Aminotransferase (units/L) | Numeric |  |
|  | SGPT | Alanine Aminotransferase (units/L) | Numeric |  |
|  | LDH | Lactic Acid Dehydrogenase (units/L) | Numeric |  |
|  | CPK | Creatine phosphokinase (units/L) | Numeric |  |
|  | NA | Sodium (mEq/L) | Numeric |  |
|  | K | Potassium (mmol/L) | Numeric |  |
|  | INR | International Normalized Ratio | Numeric |  |
| COMPLETE BLOOD COUNT | WBC | White blood cells (*1000) | Numeric |  |
|  | NEU% | Neutrophils percentage | Numeric |  |
|  | LYM% | Lymphocytes percentage | Numeric |  |
|  | MONO% | Monocytes percentage | Numeric |  |
|  | EOS% | Eosinophils percentage | Numeric |  |
|  | BASO% | Basophils percentage | Numeric |  |
|  | RBC | Red blood cells (*1000000) | Numeric |  |
|  | HGB | Hemoglobin (g/dL) | Numeric |  |
|  | HCT | Hematocrit percentage | Numeric |  |
|  | MCV | Mean Corpuscular Volume (fl) | Numeric |  |
|  | MCH | Mean Corpuscular Hemoglobin (pg) | Numeric |  |
|  | MCHC | Mean Corpuscular Hemoglobin Concentration (g/dL) | Numeric |  |
|  | RDW-CV | Red Blood Cell Distribution Width - coefficient of variation (percentage) | Numeric |  |
|  | RDW-SD | Red Blood Cell Distribution Width - standard deviation (percentage) | Numeric |  |
|  | PLT | Platelets (*1000) | Numeric |  |
|  | MPV | Mean Platelet Volume (fl) | Numeric |  |
|  | PDW | Platelet Distribution Width (percentage) | Numeric |  |
|  | PCT | Plateletcrit (percentage) | Numeric |  |
|  | P-LCR | Platelet-large cell ratio | Numeric |  |
